# The new future perspective in corneal tissue utilisation – methods of preparation and preservation

**DOI:** 10.1186/s12886-023-03048-3

**Published:** 2023-06-29

**Authors:** Martina Polachova, Magdalena Netukova, Oldrich Benada, Tomas Kucera, Vojtech Kolin, Alina-Dana Baxant, Zuzana Sirolova, Pavel Studeny

**Affiliations:** 1 Department of Ophthalmology, University Hospital Kralovske Vinohrady and 3rd Faculty of Medicine, Srobarova 1150/50, Prague 10, 100 34 Czech Republic; 2 Third Faculty of Medicine, Charles University in Prague, Ruska 87, 10000 Prague 10, Czech Republic; 3 International Eye Bank of Prague, University Hospital Kralovske Vinohrady and 3rd Faculty of Medicine, Srobarova 1150/50, Prague 10, 100 34 Czech Republic; 4 Institute of Microbiology of the Czech Academy of Sciences. Vídeňská 1083, 142 20 Prague 4 – Krč, Prague 10, 100 34 Czech Republic; 5 Institute of Histology and Embryology, First Faculty of Medicine, Charles University in Prague, Albertov 4, Prague 10, 128 00 Praha 2 Czech Republic; 6 Department of Pathology, University Hospital Kralovske Vinohrady and 3rd Faculty of Medicine, Srobarova 1150/50, Prague 10, 100 34 Czech Republic

**Keywords:** Corneal lenticule implantation, Corneal stromal lamella, Corneal tissue preparation, Effective corneal tissue utilization, Corneal tissue cryopreservation, Corneal tissue gamma-irradiation

## Abstract

**Purpose:**

The goal of our study is to find an optimal approach to the preparation and preservation of corneal stromal tissue. We want to compare different methods of corneal stromal tissue creation and storage to optimize the efficacy of this process under the conditions of an eye bank. After we find the most suitable method to create a safe high quality product, we want to prove the possibility of using a single donor cornea for more than one patient. We would also like to verify the feasibility of making more corneal lenticules after the removal of a corneal endothelium for DMEK transplantation.

**Methods:**

We provided morphological (histology, scanning electron microscope) and microbiological analysis in order to compare different methods of corneal lenticule and corneal stromal lamellae preparation and preservation. We also tested the surgical handling of the tissue to secure a safe manipulation of the tissue for clinical use. We compared two methods of corneal lenticule preparation: microkeratome dissection and femtosecond laser. As methods of preservation, we tested hypothermia, cryopreservation at -80 degrees Celsius in DMSO (dimethyl sulfoxide) and storage at room temperature with glycerol. Some intrastromal lenticules and lamellae in each group were previously irradiated with gamma radiation of 25 kGy (KiloGray).

**Results:**

Corneal stromal lamellae prepared with a microkeratome have a smoother cut – side surface compared to lamellae prepared with a femtosecond laser. Femtosecond laser preparation caused more irregularities on the surface and we detected more conglomerates of the fibrils, while lamellae made with microkeratome had more sparse network. Using femtosecond laser, we were able to make more than five lenticules from a single donor cornea.

Gamma irradiation led to damage of collagen fibrils in corneal stroma and a loss of their regular arrangement. Corneal tissue stored in glycerol showed collagen fibril aggregates and empty spaces between fibrils caused by dehydration. Cryopreserved tissue without previous gamma irradiation showed the most regular structure of the fibrils comparable to storage in hypothermia.

**Conclusion:**

Our results suggest that formation of a corneal lenticule lamellae by microkeratome results in smoother corneal lenticules, while being much cheaper than formation by femtosecond laser.

Gamma irradiation of 25 kGy caused damage of the collagen fibres as well as their network arrangement, which correlated with loss of transparency and stiffer structure. These changes impair possible surgical utilisation of gamma irradiated corneas.

Storage in glycerol at room temperature and cryopreservation had similar outcomes and we believe that both methods are appropriate and safe for further clinical use .

**Supplementary Information:**

The online version contains supplementary material available at 10.1186/s12886-023-03048-3.

## Background

The goal of our study is to find an optimal approach to the preparation and preservation of corneal stromal tissue and to prove the possibility of using one donor cornea for more recipients. In order to make this feasible in the future, a method allowing efficient and safe corneal tissue preparation and preservation is required. This leads us to study possible methods of creating donor corneal lamella in the eye bank.

Corneal transplant has been the most frequently performed tissue transplant worldwide. We still have to face a shortage of corneal tissue suitable for transplantation, given that we can only obtain the graft from cadaveric donor and the tissue has to meet strict criteria. Globally, on average, only one cornea is available for every 70 patients who do need it [1].

However, a huge amount of corneal tissue unsuitable for transplantion is being wasted worldwide every year. When we exclude the specimens with positive serology, the main reasons that the main impediments to the utilisation of these tissues are insufficient histological analysis and expiration before possible utilisation.

This leads to a long waiting time and often a worse prognosis of our patients.

In recent years, the number of performed lamellar keratoplasties has still been increasing (over 60% in comparison to the penetrating keratoplasties) [2]. During the lamellar keratoplasty, we replace just the damaged part of the patient’s cornea, which has a really positive effect on the prognosis of the patients. Compared to penetrating keratoplasty, there is a much lower risk of the graft rejection, shorter time of convalescence and, in most cases, also better visual acuity after the surgery [3].

The corneal lamellae are created from human cadaveric donor corneas by a microsurgical procedure in the tissue eye bank, which can distribute them to specialised surgical clinics [1, 4]. After the lamellae are created, the rest of the corneal tissue remains unused and is subsequently discarded. In some studies, the donor corneal endothelium was used for DMEK (Descemet’s membrane endothelial keratoplasty) and the remaining tissue was used for DALK (deep anterior lamellar keratoplasty) [5, 6], though this combined technique has not been widely performed.

To our knowledge, a wide spectrum of corneal diseases is well manageable by corneal stromal lenticule implantation. Corneal stromal lenticule can arise as a by-product from the refractive procedure ReLEx SMILE (Small Incision Refractive Lenticule Extraction), which is being performed in order to correct a refractive error [7–14]. The studies have been published, in which the corneal lenticule implantation has been used for treatment of ametropias [7], mainly hypermetropia [8, 9], presbyopia [10] and corneal ectasia at keratoconus patients [11, 12]. The other group consists of patients with severe corneal defects, ulcers [13] or corneal thinning after excessive corneal tissue removal e.g. after LASIK (laser-assisted in situ keratomileusis) surgery [14]. The corneal stromal lamella is a part of corneal stroma which, contrary to a lenticule, has a uniform thickness in all its parts. We consider that this type of tissue could be expedient, for example in cases when we want to add some tissue into an ectatic cornea.

Nevertheless, the use of the lenticules or stromal lamellae from living donors is strictly limited and there is not a sufficient amount of this tissue available in acute cases. So far, we have not been using stromal lenticules from cadaveric donors, because we do not have enough corneas even for the purposes of standard transplantation itself [1].

One of the greatest advantages of the stromal lenticule is the possibility to freeze this tissue and preserve it in a cryopreservative solution for a long time [15] and that is what we want to benefit from in our work. As far as we know, the possibility of creating a lamellae and stromal lenticule from a single donor cornea has not been described yet and we decided to study this posibility in our experiments.

Even though several studies describe different methods of corneal lenticule preservation [16], the ideal way of their preparation has not yet been defined.In our research, we followed up on previous research and did analysis of different methods. Our goal was to meet all the important criteria such as safety, with as little damage to the tissue as possible while having the possibility of long-term storage.

Our objective was to describe the most suitable method of corneal tissue preparation and preservation under tissue eye bank conditions. In cases when the corneal lamella is created elsewhere than in the clean room, such as in the operating room, terminal sterillisation is required by law. A promising possible method of terminal sterilisation seems to be gamma irradiation[16]. The impact of sterilisation by gamma-irradiation on the stromal corneal lamellae has to be further validated.

Our plan is to analyse and compare possible methods of corneal stromal tissue creation and storage, which would lead to implementation of a new product in the tissue eye bank. This would provide a suitable donor corneal tissue tailored to patients ‘ needs while reducing the amount of wasted donor corneal tissue.

In our project, we would also like to build an optimal process to be able to prepare a lenticule after obtaining a lamella for DMEK transplant from donor cornea. This would improve the efficiency of corneal tissue utilisation and increase the number of patients who would benefit from corneal lenticule implantation.

## Methods

### Used tissues

Human corneas were obtained according to the Declaration of Helsinki using the standard protocol of the Tissue Eye Bank of University Hospital Kralovske Vinohrady. We used tissues which were not suitable for transplantation due to low endothelial density, reactive serology or peripheral scarring after cataract surgery. In light of our goal, we also used corneas after previous removal of the endothelium for posterior lamellar keratoplasty which was performed at our clinic.

Tested corneas were obtained from donors with an average age of 68 ± 7 years, predominantly from women (59%). Three corneas had previously undergone a cataract surgery, but the endothelial cell count was sufficient and there was no significant scarring. The average endothelial cell count was 2543 ± 319 cells/mm2.

After deepitalisation, we made one cut of the donor cornea at the depth of 200um, which created an anterior lamella containing Bowmans membrane of the thickness of approximately 200 um and posterior lamella with the average thickness of 400um. This made it possible to produce comparable samples for further processing.

For the comparison of two different methods of stromal lamellae preparation we used paired corneas from one donor, one of them was cut with microkeratome and the other with femtosecond laser. The same approach was used for the analysis of the corneal lamella which was not irradiated before the preservation in comparison to the irradiated one. This minimised the bias depending on possible different donor cornea characteristics. At the end of the experiments, we tested the possibility of creating multiple stromal lamellae from a single donor cornea by femtosecond laser. The first cut started at the depth of 100um and after the removal of the first lamella with the thickness of 100um, we continued with deeper cuts of lamellae with the thickness of 100 um.

### Corneal tissue preservation

All procedures, except for gamma irradiation, were performed under appropriate conditions at the Tissue Eye Bank (Department of Ophthalmology, University Hospital Královské Vinohrady and 3rd Faculty of Medicine in Prague).

We compared fresh control corneas stored in Eusol-C © (Alchimia srl, Italy) at 4 °C to following groups: 1. corneal stromal tissue stored in hypothermia at 4 °C in glycerol (Glyo-on, Alchimia, Italy); *n* = 6, 2. gamma-irradiated corneal tissue (25 kGy, Bioster, Veverská Bitýška, Czech Republic) stored in glycerol; *n* = 6, 3. corneal stromal tissue cryopreserved in 10% DMSO (dimethylsulfoxide); *n* = 6, 4. gamma-irradiated corneal tissue (25 kGy, Bioster, Veverská Bítýška, Czech Republic) subsequently cryopreserved in 10% DMSO at -80 °C; *n* = 6.

Half of the specimens from each group were analysed by histological and SEM (scanning electron microscope) analysis. The images were assessed by two independent experienced histologists from the 1st and the 3rd Faculty of Medicine in Prague.

### FS laser versus microkeratome

In the first step, we decided to verify the differences between the surface of the corneal stromal lamellae made by femtosecond laser and microkeratome. The corneas which were not suitable for transplantation, stored in Eusol-C © at 4 °C, were cut into 10 lamellae in each group. Using the microkeratome Moria and FS laser (Ziemer femto LDV Z8) we created corneal lamellae in the diameter of 8 mm. Then we stored them in Eusol-C © at 4 °C and transported them to morphological analysis. In order to avoid bias resulting from the intercorneal variations, we used both paired corneas from each donor, one was cut with microkeratome and the second one with femtosecond laser. The samples were then analysed by two independent experienced histologists.

### SEM analysis (scanning electron microscope)

We followed the standard tissue preparation protocol for scanning electron microscopy analysis. All samples were pre-washed in PBS (phosphate-buffered saline) buffer and fixed in 2.5% glutaraldehyde in PBS for 2 h at room temperature (RT) and overnight at 4 °C. Fixed samples were extensively washed with PBS buffer (3x, 20 min each) at RT and postfixed for 2 h in aqueous 1% OsO_4_ at RT.

The postfixed samples were washed in copious amounts of redistilled water (3x, 20 min, each) and dehydrated in the graded ethanol series (25%, 50%, 75%, 90%, 96%, 100%, and 100%; 20 min each) at RT. Dehydrated samples in 100% ethanol were cooled to 4 °C and critical point dried (K850 Critical Point dryer, Quorum Technologies Ltd, East Sussex, United Kingdom). Dry specimens were mounted by high-purity conductive double-sided adhesive carbon tabs (EM-Tec CT12, 12 mm) onto standard 12 mm aluminum stubs (G301Z Pin stubs). Subsequently, they were coated with 3 nm platinum in a high-resolution sputter coater (Q150T SE, Quorum Technologies Ltd, East Sussex, United Kingdom). The final samples were analysed in a FEG scanning electron microscope FEI Nova NanoSEM450 (FEI, Brno, Czech Republic) at 3 kV using SE, ETD, TLD, and CBS detectors (secondary electrons detectors and back-scattered electrons detectors). The image acquisition was performed in 16-bit TIFFs with subsequent conversion to 8-bit TIFFs in the original FEI software.

The scans were analysed by two independent experts, a histologist and a pathologist, who described the morphological changes of the specimens in comparison to the reference fresh cornea.

### Histological analysis

The material was processed using a standard histological technique with formalin fixation and paraffin embedding. Parrafin blocks were cut with a microtome, the preparations were stained with hematoxyline and eosin. All samples were then analysed by two independent experts, a histologist and a pathologist, who described the morphological changes of the specimens in comparison to the reference fresh cornea.

## Results

### Electron microscope and macroscopy

#### Preparation technigues

**Fig.1:**
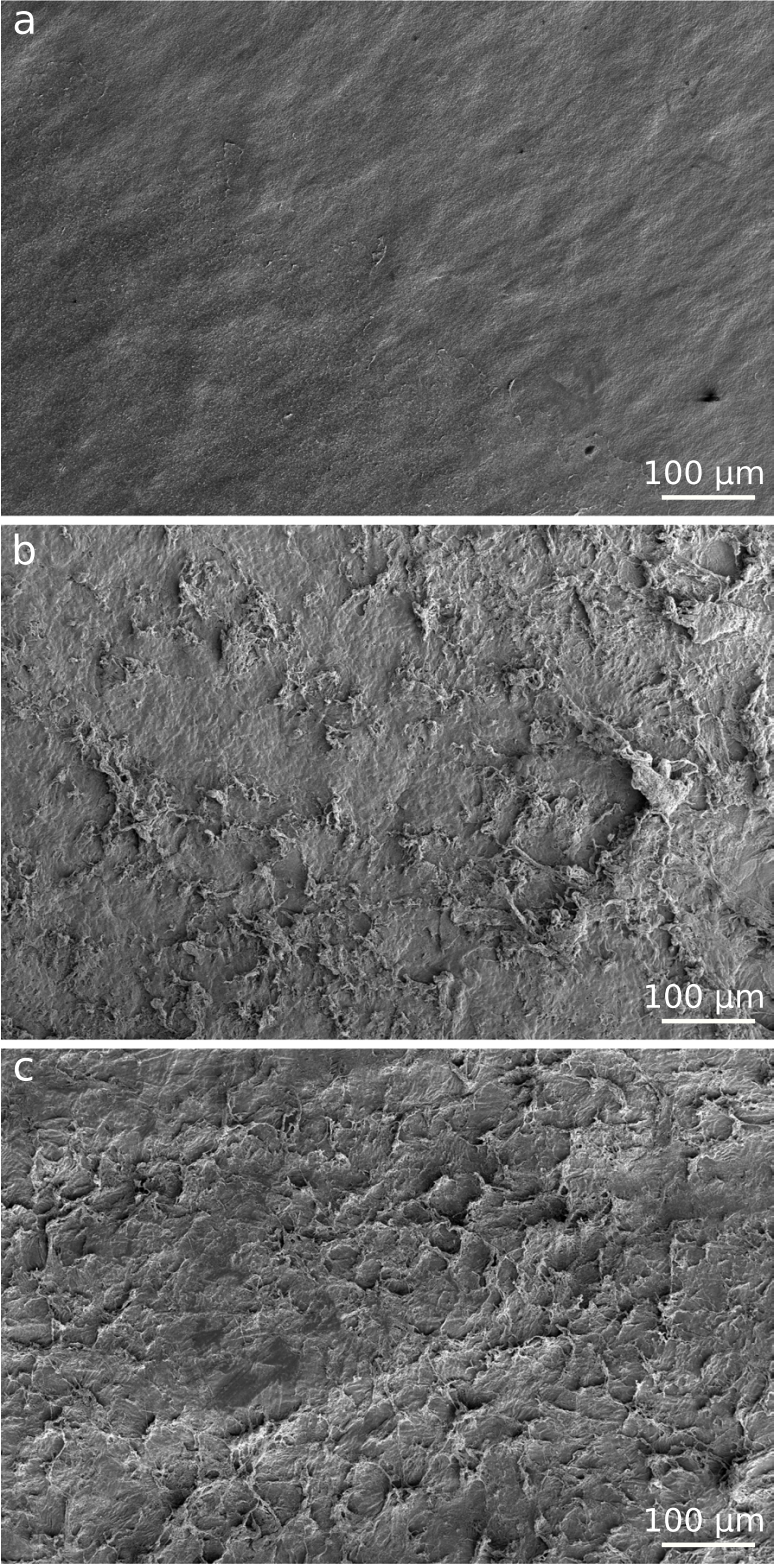
(scanning electron microscope): **a**—surface of a corneal lamellae made by microkeratome. **b** – surface of a corneal lamellae made by femtosecond laser. **c**—surface of a corneal lenticulae made by femtosecond laser

The scanning electron microscopy confirmed that corneal stromal lamellae prepared by microkeratome had smoother cut-side surface than the ones prepared by femtosecond laser (Fig. 1A) Femtosecond laser preparation caused more irregularities on the interface (Fig. 1B,C). In histological images, we detected more conglomerates of the fibrils, while lamellae prepared by microkeratome had sparser network. Using femtosecond laser we were able to create more than five lenticules from a single donor cornea, which were easy to manipulate by standard surgical procedures.

### Preservation methods

**Fig. 2:**
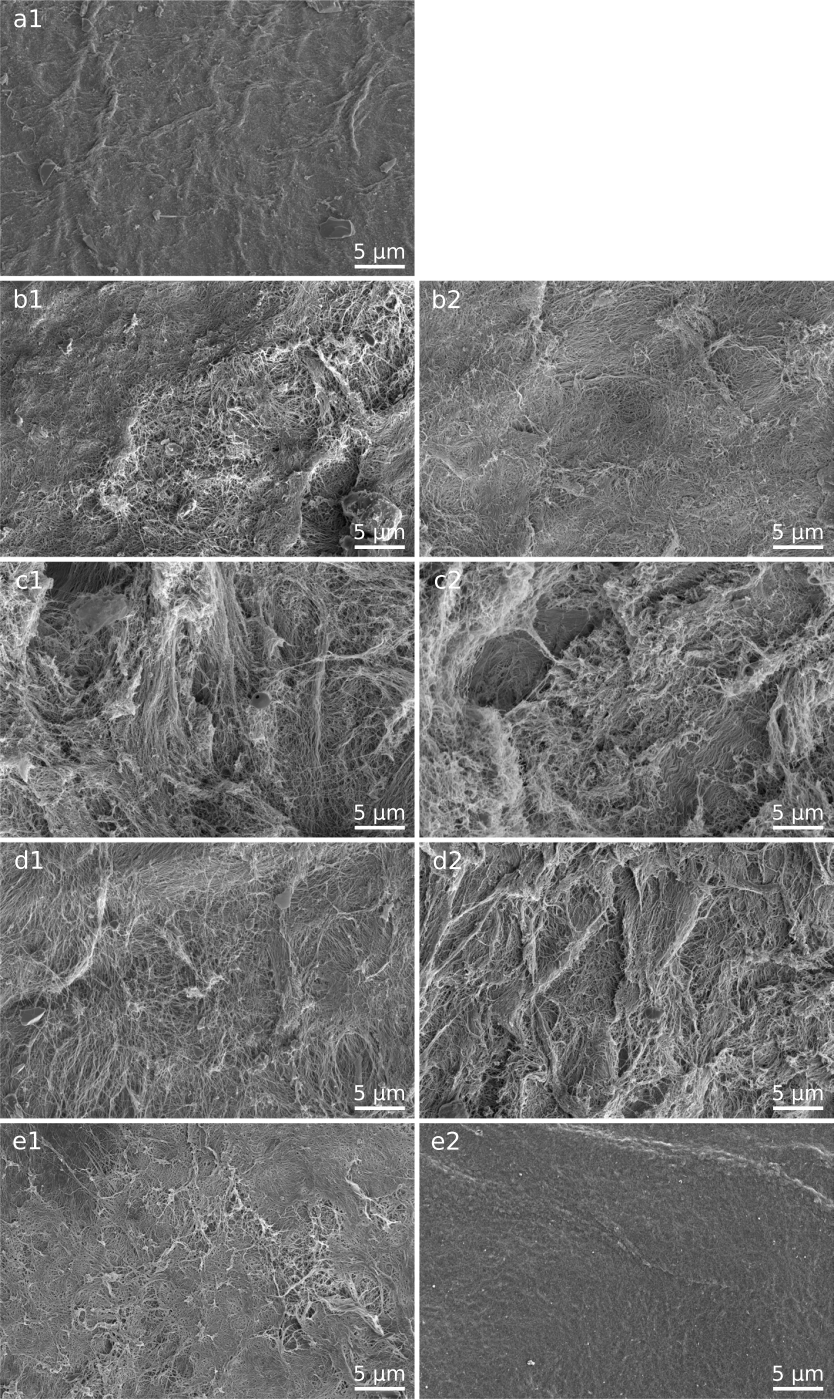
(scanning electron microscope): **a**1 – fresh cornea **b**1 – gamma-irradiated corneal lamella (anterior stroma) stored in glycerol. **b**2—gamma-irradiated corneal lamella (posterior stroma) stored in glycerol **c**1 – gamma-irradiated corneal lamella (anterior stroma) cryopreserved in DMSO **c**2 – gamma-irradiated corneal lamella (posterior stroma) cryopreserved in DMSO **d**1 – non-irradiated corneal lamella (anterior stroma) stored in glycerol **d**2 – non-irradiated corneal lamella (posterior stroma) stored in glycerol **e**1 – non-irradiated corneal lamella (anterior stroma) cryopreserved in DMSO **e**2 – non-irradiated corneal lamella (posterior stroma) cryopreserved in DMSO

Corneal stromal tissue stored in hypothermia at 4 °C in glycerol stayed macroscopically transparent with signs of dehydration (Supplementary Figs. 5,6). In electron microscopic analysis we detected cavitation bubbles between the collagen fibres, which correlated with the degree of dehydration. There were smaller aggregates of the fibrils, which were in bundles aligned in parallel (Fig. 2D). The fibrils showed less signs of damage in comparison with gamma-irradiated tissue and the fibrils formed more regular network. Corneal stromal tissue stored in glycerol after irradiation revealed fibres “baked” into each other (Fig. 2B). Macroscopically, the tissue was yelowish and lost its transparency (Supplementary Figs. 1, 2).

Corneal stromal tissue cryopreserved in DMSO without subsequent irradiation was macroscopically clear without significant loss of transparency while preserving the elasticity for easy surgical manipulation (Supplementary Figs. 7,8). Electron microscopy revealed regullar structure and parallel alignment of the collagen fibrils (Fig. 2E). In contrast with cryopreserved lamellae and lenticullae which were irradiated, there were no areas of fibril breakup which we found in irradiated tissue. In irradiated samples stored by cryopreservation, the parallel lamellar alignment of the fibrils was destroyed and we found places of collagen network disintegration (Fig. 2C). Macroscopically, these tissues have lost their transparency and normal shape which did not allow surgical utilisation (Supplementary Figs. 3,4).

Electron microscopy analysis of the corneal lamellae processed by above-described methods is summarised in Fig. 2 in separate pictures. Analogous macroscopic findings are demonstrated in Supplementary Figs. 1–8.

### Histological analysis

**Fig. 3:**
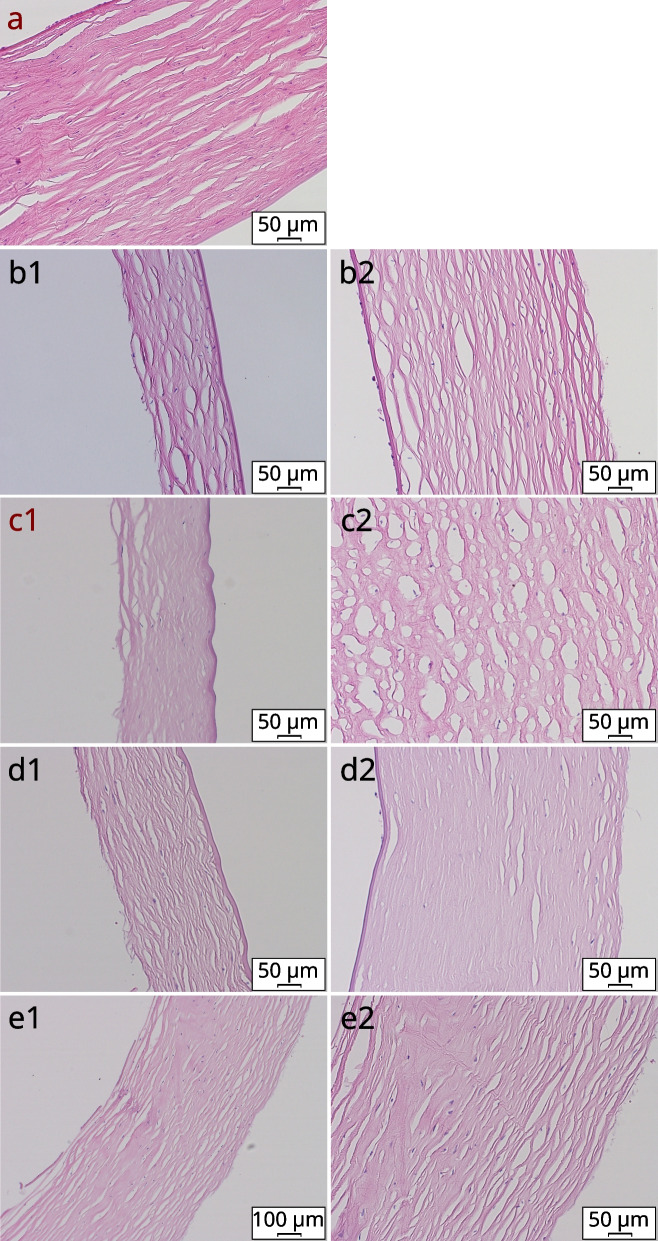
(histological analysis): **a** – fresh cornea **b**1 – gamma-irradiated corneal lamella (anterior stroma) stored in glycerol **b**2 – gamma-irradiated corneal lamella (posterior stroma) stored in glycerol **c**1 – gamma-irradiated corneal lamella (anterior stroma) cryopreserved in DMSO **c**2 – gamma-irradiated corneal lamella (posterior stroma) cryopreserved in DMSO **d**1 – non-irradiated corneal lamella (anterior stroma) stored in glycerol **d**2 – non-irradiated corneal lamella (posterior stroma) stored in glycerol **e**1 – non-irradiated corneal lamella (anterior stroma) cryopreserved in DMSO **e**2 – non-irradiated corneal lamella (posterior stroma) cryopreserved in DMSO

Histological analysis corellated with the macroscopic and electron microscopic findings. We compared the results with our reference specimen – fresh cornea (Fig. 3A). Gamma-irradiated corneas stored in glycerol showed condensed stromal structure, collagen fibres disintegration as well as loss of cell nuclei stainability. Corneal stromal lamellae preserved in the same manner reported the greatest disintegration of fibrils in the posterior stroma, whereas the anterior stroma had distinct retraction artifacts (Fig. 3B).

Whole corneas stored in glycerol without subsequent gamma irradiation had fried and dehydrated stroma, while corneal stromal lamellae had good interfibrillar integrity (Fig. 3D).

Full-thickness corneas cryopreserved in DMSO after gamma irradiation showed loss of stromal structure, decreased stainability of the tissue and a vast amount of retraction artifacts. In the anterior lamellae, there were large interfibrilar spaces due to retraction. The posterior stromal lamellae revealed high vulnerability of the elements (Fig. 3C).

Cryopreserved corneas in DMSO had no retraction artefacts, which corresponds to low vulnerability of the tissue, histologically it has the highest similarity to the fresh corneas. Stromal lamellae revealed a good integrity of collagen fibres and good stainability of cell nuclei (Fig. 3E).

The structural analysis is summarised in Table 1.

**Table 1:** Structural analysis of studied tissues

Table 1	**Macroscopy**	**Histology**	**SEM**
Fresh cornea	Transparent	Regular parallel fibrils No retraction artefacts	Parallel collagen fibres No disintegration
DMSO -80 °C	Transparent Elastic structure	Good collagen fibres integrity Good nuclear stainability No retraction artefacts	Regular structure Parallel fibres alignment
GI + DMSO -80 °C	Yellowish with loss of transparency Stiff structure	Loss of stromal structure Decreased nuclear stainability Retraction artefacts	Areas of collagen fibres break-up Irregular disintegrated collagen network
Glycerol 4 °C	Dehydrated Transparent	Fryed and dehydrated stroma Good interfibrillar integrity in stromal lamellae	Regular collagen network Smaller aggregates of the fibrils in parallel alignment
Glycerol + GI	Yellowish Loss of transparency Stiff structure	Fryed stromal structure Collagen disintegration (mainly in posterior stroma) Loss of nuclear stainability	Collagen fibres disintegration Loss if regular network Collagen fibres conglomerates

*GI* gamma irradiation, *DMSO* Dimethyl sulfoxide

## Discussion

The goal of our study was to evaluate the possible methods of corneal tissue preparation and preservation which could maximize the usability and accessibility of donor corneal tissue.

With regard to preparation of corneal lamellae, both methods – femtosecond laser and microkeratome seem to be safe and well feasible. The biggest advantage of femtosecond laser was the feasibility of creating even five or more stromal lenticules or lamellae from one donor cornea. The femtosecond laser software also allows the setting of the intended depth and thickness of the lamella. In the future, this could allow storage of lamellae with different characteristics, such as their thickness and collagen network density depending on the depth of the cut in corneal stroma. The optimal stromal lamella could then be selected for the customised needs of the patients. This could also be favorable in order to prepare more transplants from a single donor cornea and allows their use for several patients with corneal defects or thinning of a different extent.

On the other hand, microkeratome preparation excelled in smooth surface of the incision with no irregularities at the interface. These lamellae had a sparser network with no ultrastructural conglomerates of collagen fibrils.

Both methods allowed to produce a corneal lenticule or a stromal lamela from the donor cornea, from which a lamella had previously been removed for DMEK transplantation.

Obviously, the entire preparation process enabling creation of multiple stromal transplants from a single donor cornea has to be performed in the clean room of a tissue eye bank and then distributed to patients. As far as we know, the use of a single donor cornea for more recipients is allowed by European legislation. However, the exact regulations may differ in different countries.

As to preservation, we focused on the structural and ultrastructural characteristics of corneal tissue preserved by different methods.

Prior to the experiments, we believed that sterilisation by gamma irradiation could be a safe way to prepare donor corneal tissue easily available for the eye departments with no availability of a tissue bank. However, the gamma-irradiated corneal tissue revealed serious structural damage and seems to be inappropriate for corneal stromal tissue sterilisation.

Macroscopic analysis of lamellae and lenticulae irradiated by gamma irradiation showed deformation and yellowish colour with subsequent loss of their transparency. The structure became more rigid with loss of the elasticity. This correlated with histological and electron microscopic analysis, which described damage of collagen fibrils in corneal stroma and loss of their regular arrangement. In ultrastructural analysis, we reached the same results as previously provided in a study by Chae et al. where the authors describe significantly lower collagen fibre density as well as collagen fibre thickness after gamma irradiation in comparison to the fresh corneas. In contrast with our study, they found no significant differences in transparency and elastic modulus between irradiated and fresh samples [17]. This could however be influenced by a different dose of gamma irradiation, as the authors used a source of 17–23 kGy.

The glycerol storage has already been described by several studies, which came to conclusion that it is a safe method the storage of corneal tissue [16, 18]. Our results resembled the findings of Liu et al., who described partial edema of collagen fibers, fused cavitation bubbles, and few keratocytes. Their TEM analysis showed that the mean number of corneal collagen fibrils in the glycerol group was lower than in the in control group, but the diameter of the fibrils was almost unchanged. They attributed this to an incomplete dehydration or a short preservative period [18]. In our analysis, we also noticed slight differences between the ultrastructure of glycerol-stored whole corneas and stromal lamellae. The whole corneas had fryed and dehydrated stroma, while corneal stromal lamellae had good interfibrillar integrity.

The most simillar structure compared to fresh donor corneas was found in cryopreserved corneas stored in DMSO in -80 °C. The tissue remained transparent and elastic thanks to the regular collagen network and no fibrillar damage.

Corneas stored in glycerol did not lose their transparency, however there were distinct changes caused by glycerol dehydration.

From our experiments, we can deduce that cryopreservation is a safe and appropriate method allowing long-term storage of the corneal stromal tissue. As far as we know, it has already been the most frequently used method in corneal lenticule storage and its successful reimplantation was proved in animal model as well as in human patients [9, 18]. Studies have proved that cryopreserved lenticules maintained their structural integrity of collagen fibres and cell viability [19]. We expect that the results with corneal stromal lamellae would be very similar to the experiments with stromal lenticules as the character of the tissue is the same, differing chiefly by the shape of the transplant.

However, cryopreservation is appropriate only in cases when the viable endothelium is not required, because despite some successful cryopreserved corneal grafts, freezing has been shown to damage the endothelium [20, 21].

We are aware of the limitations of our study, such as small number of the samples. We were also limited by the gamma irradiation dose of 25 kGy, as it could be too high to aggravate the corneal structural changes after irradiation. Due to the analytical procedures, there was no possibility to examine the same cornea before and after the preservation process.

In the future, we may expect reducing quantity requirements of donor corneal tissue thanks to new biosynthetic materials. New biosynthetic materials for the arteficial keratoprotesis are still being developed and have been studied in recent years. Even though they seem to be a solution for patients where conventional cornea transplantation is not possible, they still have limitations and complications. Finding the optimal approach allowing the expansion of their clinical use requires further adequate study and evaluation [22, 23].

## Conclusion

In conclusion, we provided the macroscopic and ultrastructural analysis of corneas, corneal lamellae and corneal stromal lenticules prepared and preserved by different methods. With regard to methods of preparation, microkeratome and femtosecond laser appear to be safe and feasible methods in corneal stromal lamellae creation. Both have their unique advantages, however, when creating multiple lamellae or lenticules from a single donor, the femtosecond laser excelled. We verified the feasibility of making more corneal lamellae from a single donor cornea. Moreover, we proved the practicability of obtaining a stromal lamella after the removal of endothelial lamella for standard DMEK transplantation under tissue eye bank conditions. To our knowledge, this has not been further described, yet. In the future, different settings of the preparation parameters could also enable to use a customised donor stromal corneal implant for a specific patient depending on the extent of their corneal disease.

Cryopreservation seems to be the most favorable method for our purposes, as it allows us the safe long-term storage of the corneal tissue in cases when living endothelium is not required. It could theoretically help us to reduce the number of discarded corneas due to the expiration date or due to the low endothelial density, as these tissues can now be stored in the form of frozen lamellae or lenticullae for further use. Compliance with our process facilitates the safe and efficient use of a single donor cornea for more than one patient which helps to optimize the use of donor cornea while minimizing the amount of wasted tissue.

In order to be able to induct our approach into clinical praxis, more data is still required.

## Supplementary Information

**Additional file 1: Supplementary figure 1.** gamma-irradiated cornea in glycerol.

**Additional file 2: Supplementary figure 2.** gamma-irradiated lamella in glycerol.

**Additional file 3: Supplementary figure 3.** gamma-irradiated cornea cryopreserved in DMSO.

**Additional file 4: Supplementary figure 4.** gamma-irradiated corneal lamella cryopreserved in DMSO.

**Additional file 5: Supplementary figure 5.** non-irradiated cornea stored in glycerol.

**Additional file 6: Supplementary figure 6.** non-irradiated corneal lamella stored in glycerol.

**Additional file 7: Supplementary figure 7.** non-irradiated cornea cryopreserved in DMSO.

**Additional file 8: Supplementary figure 8.** non-irradiated corneal lamella cryopreserved in DMSO.

## Data Availability

All data supporting our findings can be shared upon request to the corresponding author (martina.polachova@fnkv.cz), although the majority is contained within the manuscript.

## Abbreviations

DALK: Deep anterior lamellar keratoplasty
DMEK: Descemet’s membrane endothelial keratoplasty
DMSO: Dimethyl Sulfoxide
LASIK: Laser assisted in situ keratomileusis
kGy: KiloGray
PBS: Phosphate buffered saline
ReLEx SMILE: Small Incision Refractive Lenticule Extraction
RT: Room temperature
SEM: Scanning electrone microscope
