# Supplementary figures and images for: The new future perspective in corneal tissue utilisation – methods of preparation and preservation
Source: BMC Ophthalmol. 2023 Jun 29;23:294. doi: 10.1186/s12886-023-03048-3 (PMC10308633; doi:10.1186/s12886-023-03048-3)

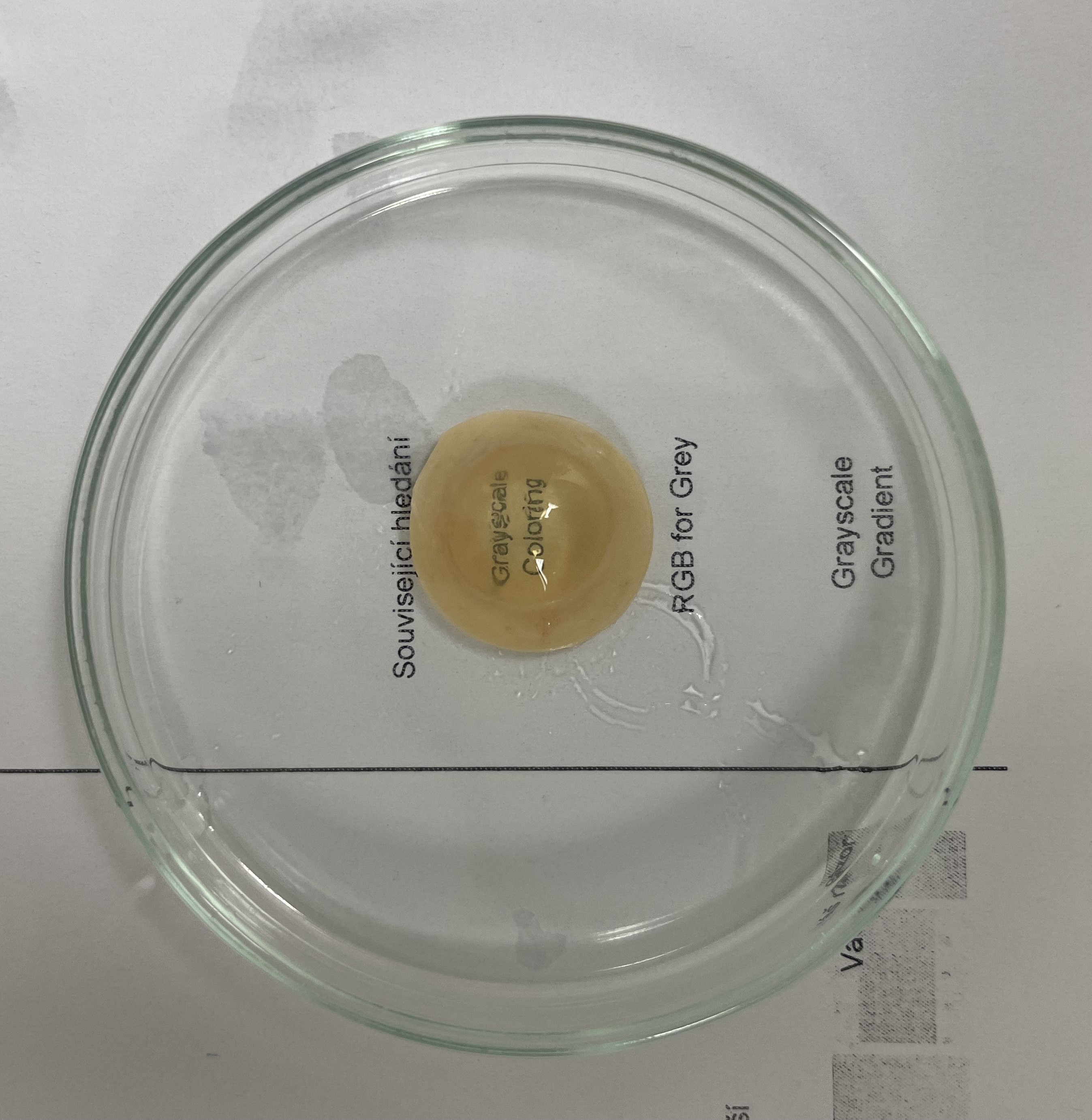

Supplement: Supplementary file 1 — Additional file 1: Supplementary figure 1. gamma-irradiated cornea in glycerol. [file 12886_2023_3048_MOESM1_ESM.jpg]

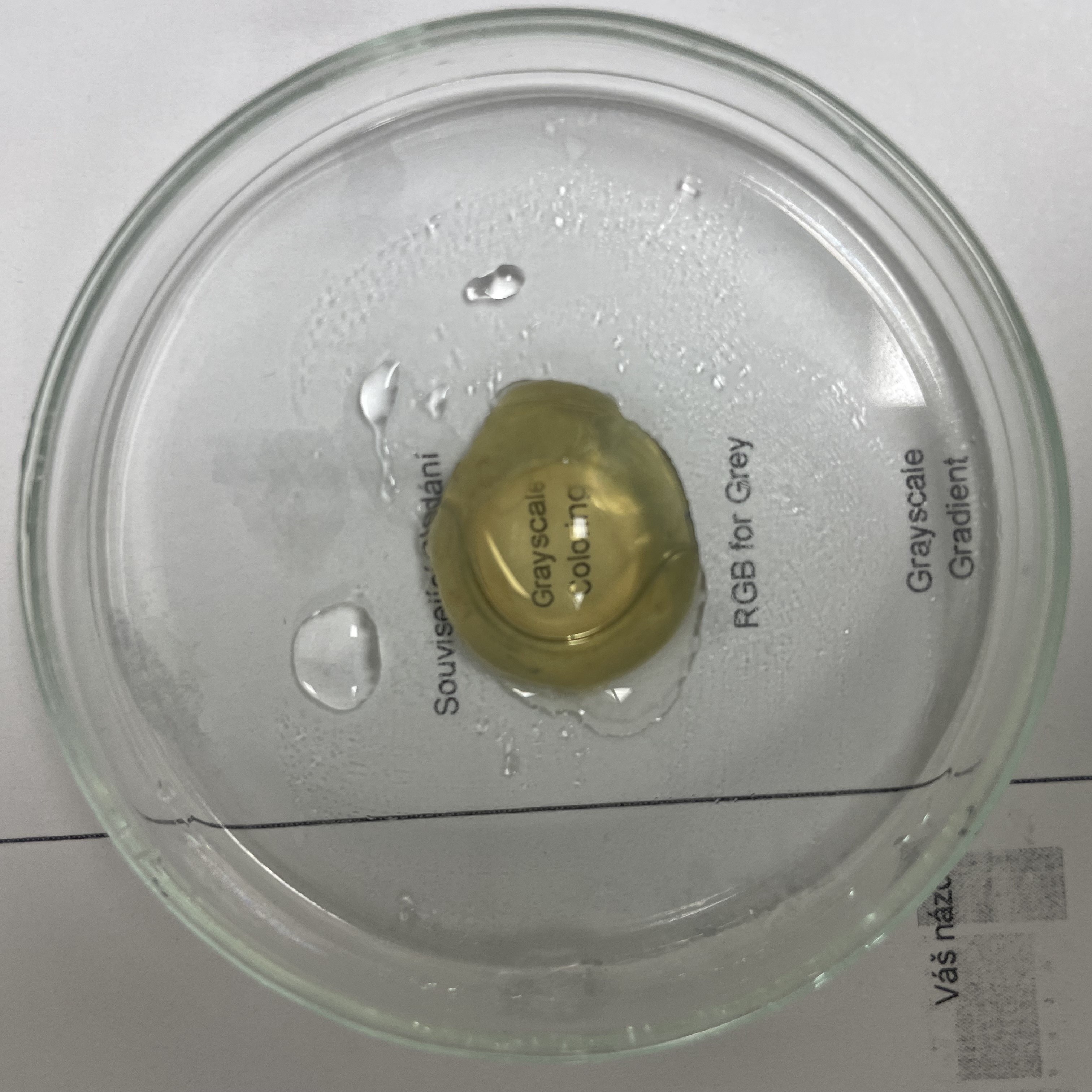

Supplement: Supplementary file 2 — Additional file 2: Supplementary figure 2. gamma-irradiated lamella in glycerol. [file 12886_2023_3048_MOESM2_ESM.jpg]

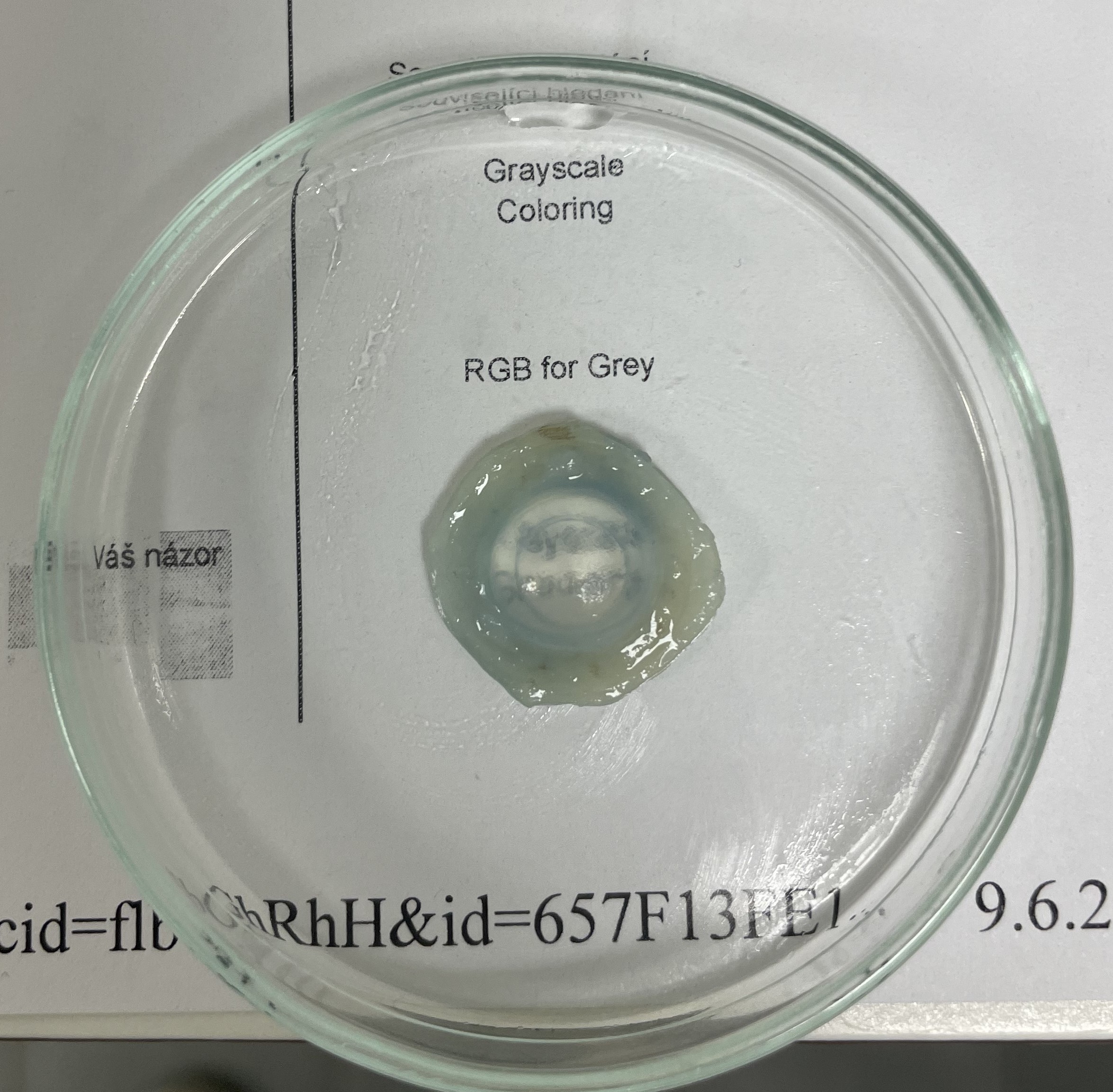

Supplement: Supplementary file 3 — Additional file 3: Supplementary figure 3. gamma-irradiated cornea cryopreserved in DMSO. [file 12886_2023_3048_MOESM3_ESM.jpg]

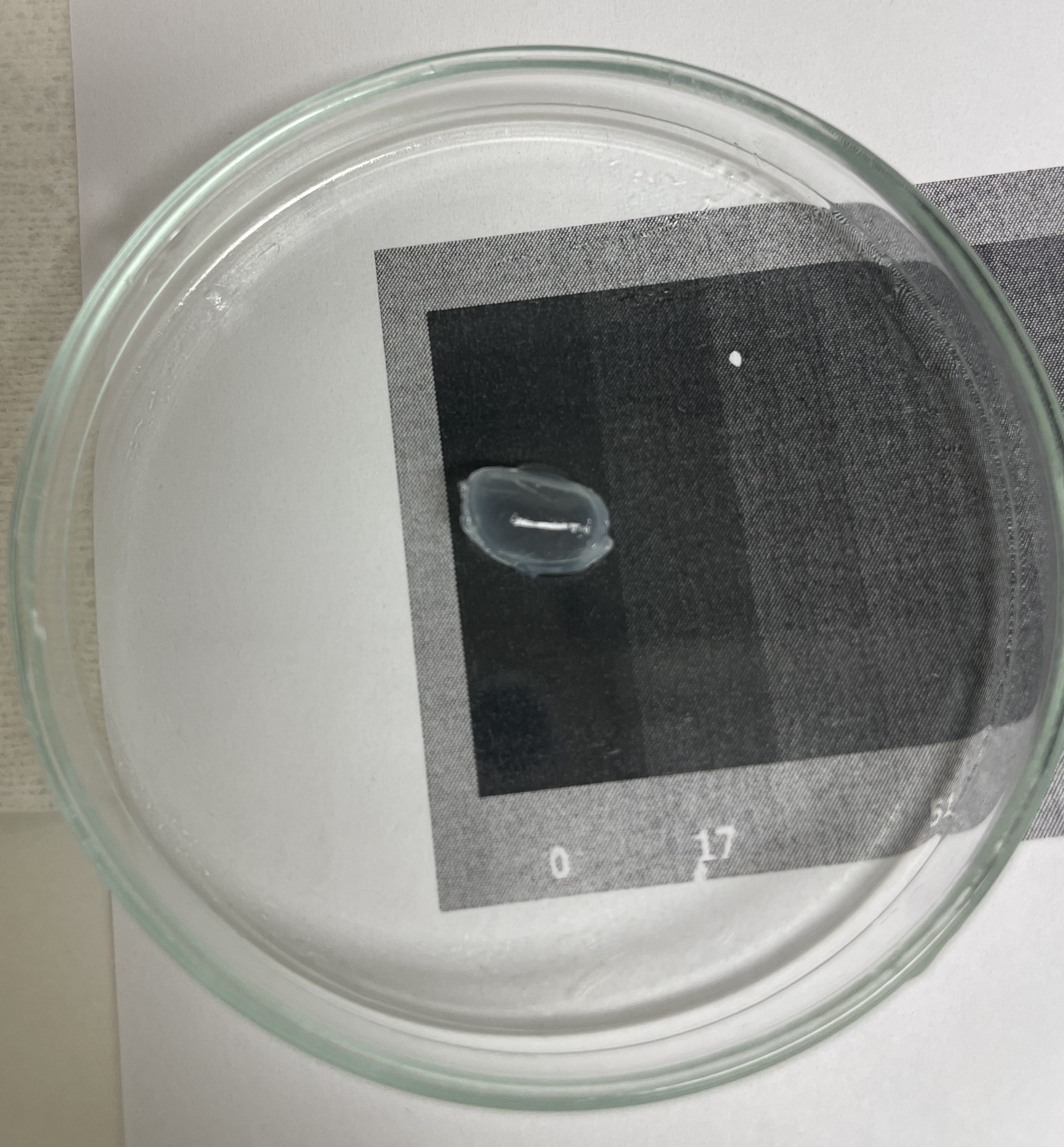

Supplement: Supplementary file 4 — Additional file 4: Supplementary figure 4. gamma-irradiated corneal lamella cryopreserved in DMSO. [file 12886_2023_3048_MOESM4_ESM.jpg]

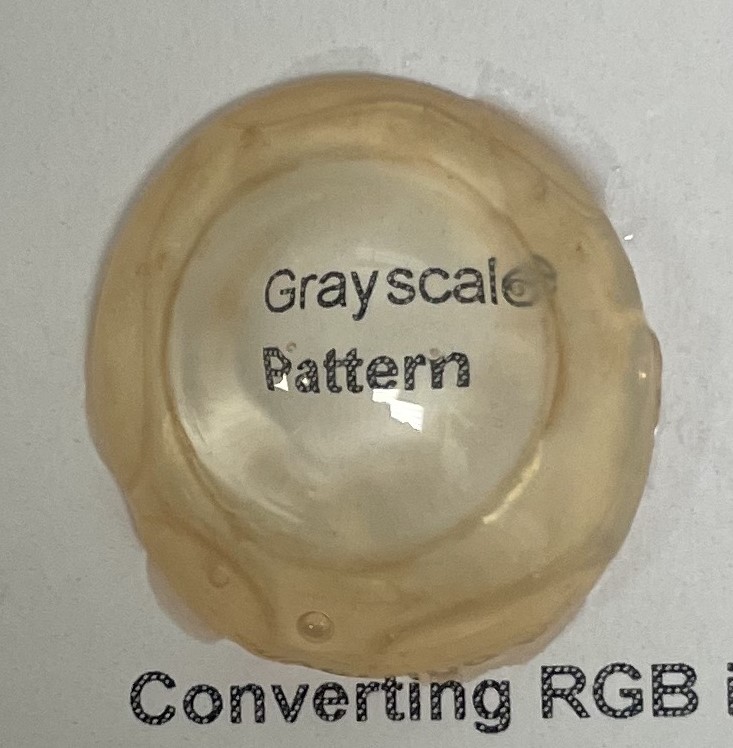

Supplement: Supplementary file 5 — Additional file 5: Supplementary figure 5. non-irradiated cornea stored in glycerol. [file 12886_2023_3048_MOESM5_ESM.jpg]

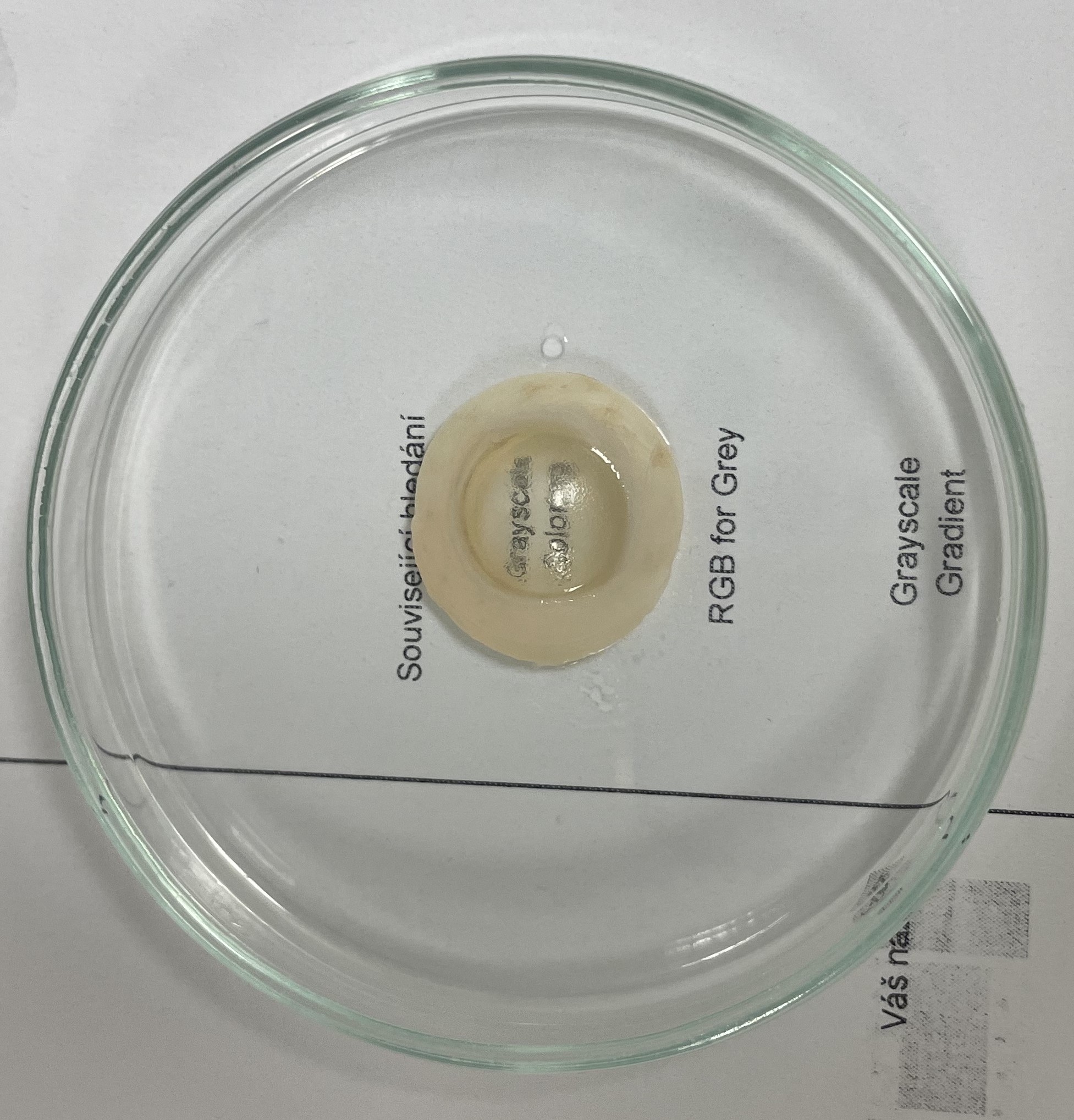

Supplement: Supplementary file 6 — Additional file 6: Supplementary figure 6. non-irradiated corneal lamella stored in glycerol. [file 12886_2023_3048_MOESM6_ESM.jpg]

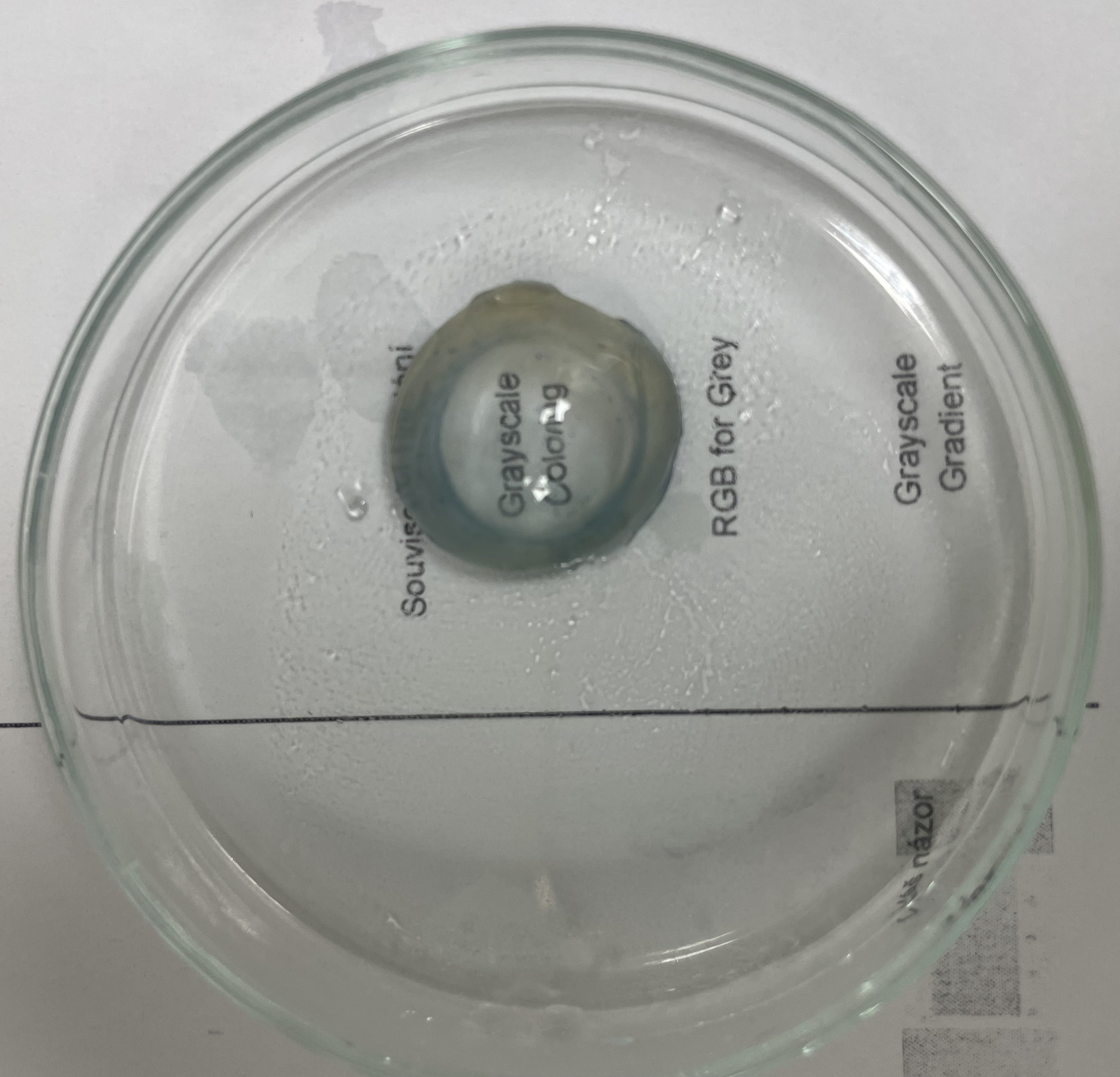

Supplement: Supplementary file 7 — Additional file 7: Supplementary figure 7. non-irradiated cornea cryopreserved in DMSO. [file 12886_2023_3048_MOESM7_ESM.jpg]

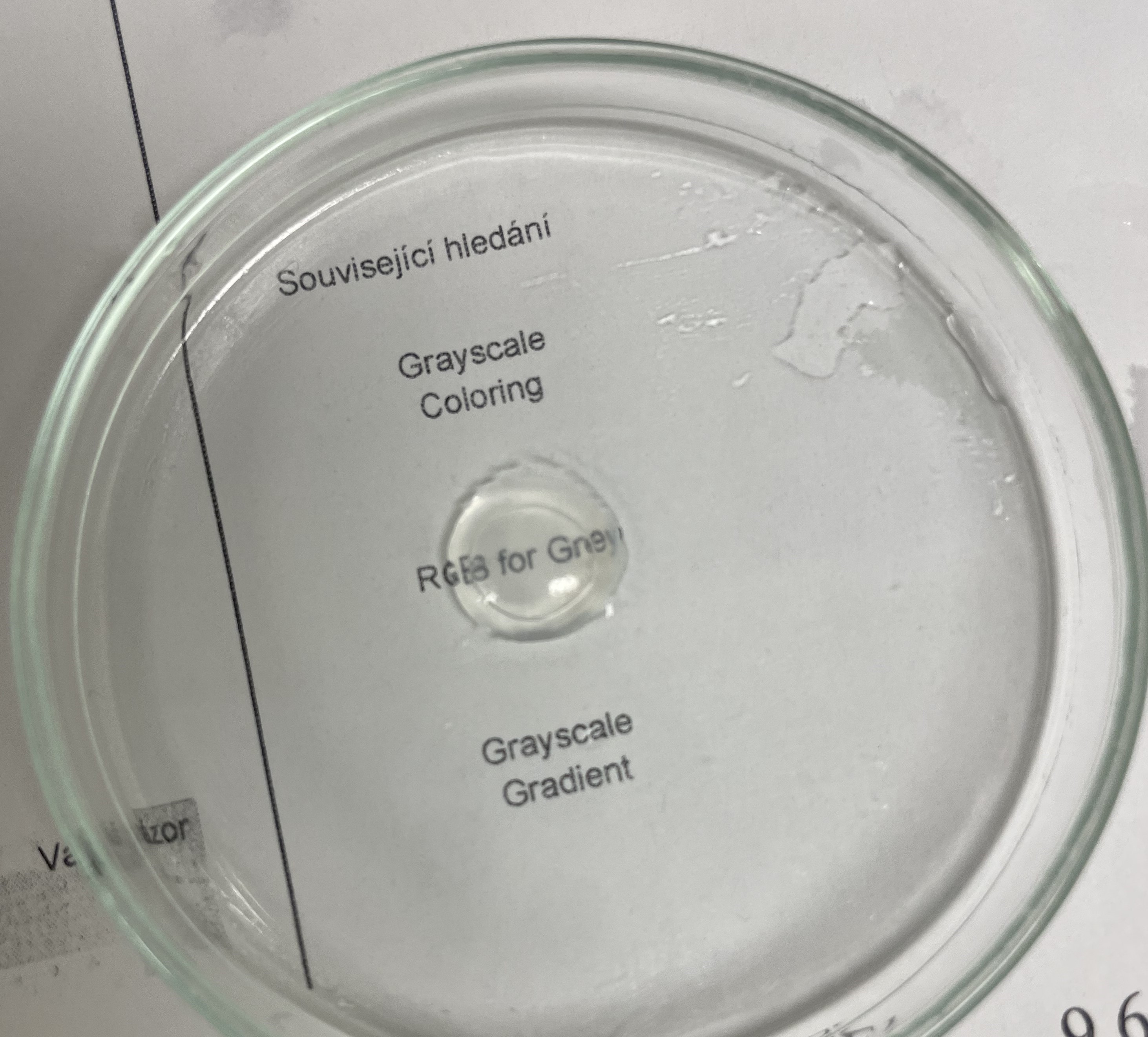

Supplement: Supplementary file 8 — Additional file 8: Supplementary figure 8. non-irradiated corneal lamella cryopreserved in DMSO. [file 12886_2023_3048_MOESM8_ESM.jpg]
